# Use of antenatal corticosteroids prior to preterm birth in four South East Asian countries within the SEA-ORCHID project

**DOI:** 10.1186/1471-2393-8-47

**Published:** 2008-10-16

**Authors:** Porjai Pattanittum, Melissa R Ewens, Malinee Laopaiboon, Pisake Lumbiganon, Steven J McDonald, Caroline A Crowther, The SEA-ORCHID Study Group

**Affiliations:** 1 Department of Biostatistics and Demography, Khon Kaen University, Khon Kaen 40002, Thailand; 2 Discipline of Obstetrics and Gynaecology, University of Adelaide, SA 5006, Australia; 3 Department of Obstetrics and Gynaecology, Khon Kaen University, Khon Kaen 40002, Thailand; 4 Australasian Cochrane Centre, Monash University, Melbourne, VIC 3168, Australia

## Abstract

**Background:**

There is strong evidence supporting the use of antenatal corticosteroids in women at risk of preterm birth to promote fetal lung maturation and reduce neonatal mortality and morbidity. This audit aimed to assess the use of antenatal corticosteroids prior to preterm birth in the nine hospitals in four South East Asian countries participating in the South East Asia Optimising Reproductive Health in Developing Countries (SEA-ORCHID) Project.

**Method:**

We reviewed the medical records of 9550 women (9665 infants including 111 twins and two triplets) admitted to the labour wards of nine hospitals in four South East Asian countries during 2005. For women who gave birth before 34 weeks gestation we collected information on women's demographic and pregnancy background, the type, dose and use of corticosteroids, and key birth and infant outcomes.

**Results:**

Administration of antenatal corticosteroids to women who gave birth before 34 weeks gestation varied widely between countries (9% to 73%) and also between hospitals within countries (0% to 86%). Antenatal corticosteroids were most commonly given when women were between 28 and 34 weeks gestation (80%). Overall 6% of women received repeat doses of corticosteroids. Dexamethasone was the only type of antenatal corticosteroid used.

Women receiving antenatal corticosteroids compared with those not given antenatal corticosteroids were less likely to have had a previous pregnancy and to be booked for birth at the hospital and almost three times as likely to have a current multiple pregnancy. Exposed women were less likely to be induced and almost twice as likely to have a caesarean section, a primary postpartum haemorrhage and postpartum pyrexia.

Infants exposed to antenatal corticosteroids compared with infants not exposed were less likely to die. Live born exposed infants were less likely to have Apgar scores of < 7 at five minutes and less likely to have any lung disease.

**Conclusion:**

In this survey the use of antenatal corticosteroids prior to preterm birth varied between countries and hospitals. Evaluation of the enablers and barriers to the uptake of this effective antenatal intervention at individual hospitals is needed.

## Background

Infants born preterm are at high risk of neonatal lung disease and associated complications [1]. Respiratory distress syndrome (RDS), a consequence of immature lung development, is the primary cause of early neonatal mortality [2] and contributes to significant immediate [1] and long-term morbidity in survivors [3]. Antenatal corticosteroid treatment for women at risk of very preterm birth before 34 weeks gestation is one of the most effective treatments for the prevention of RDS, reducing neonatal mortality and morbidity [4]. This is of importance for developing countries where resources are scarce and it is often difficult to provide expensive treatments such as neonatal care.

Although evidence of benefit for the use of antenatal corticosteroids has been available for many years [5,6], widespread adoption of this treatment into routine clinical practice was significantly delayed and implementation of their use remained suboptimal in developed countries for many years [7]. There is limited information as to how well this practice has been implemented in developing countries.

### Objectives of this review

The aim of this audit was therefore to assess the use of antenatal corticosteroids for women who gave birth at less than 34 weeks gestation in the nine hospitals across four South East Asian countries within the South East Asia Optimising Reproductive Health in Developing Countries (SEA-ORCHID) Project [8]. Information was collated on which women received corticosteroids, their sociodemographics and obstetric history, the type, amount and reason for use of corticosteroids, the birth details and infant health outcomes.

## Methods

### Setting

Nine hospitals participating in the SEA-ORCHID project in four South East Asian countries namely Indonesia, Malaysia, the Philippines and Thailand were audited [9]. Seven of the nine hospitals were tertiary (university and regional) referral institutions with regional referrals of women with a high risk pregnancy and two hospitals were provincial or district institutions. Delivery care models included obstetric specialists or a multidisciplinary approach with midwives (including nurses with midwifery qualifications). In all hospitals obstetric specialists and caesarean section facilities were available and doctors and/or midwives (including nurses with midwifery qualifications) conducted normal vaginal births.

The SEA-ORCHID project settings and methods have been described elsewhere [8]. The study was approved as an audit by the local ethics committee of each hospital and the administering institution in Australia. Individual patient consent was not therefore sought.

### Procedure

Previously, as part of the SEA-ORCHID baseline data collection we completed a prospective audit of the medical records of 9550 women (9665 infants including 111 twins and two triplets) admitted to the labour wards at the nine participating SEA-ORCHID hospitals during 2005. At five of the participating hospitals medical records were audited on a consecutive basis until a sample of 1000 was reached. To reduce the risk of bias the four largest participating hospitals randomly sampled medical records using various ratios to ensure a sample of 1000 was reached over the three month data collection period.

For this current audit, staff at each hospital were trained to use pre-established data extraction forms to prospectively audit the medical records of the 290 women who gave birth at < 34 weeks regardless of whether they were given antenatal corticosteroids or not (Table 1). Data from each country was sent to Khon Kaen University by post where it was manually entered into a Microsoft Access 2003 database. The database minimised transcription errors by performing validation checks to detect discrepancies and missing data.

**Table 1:** Percentage distribution of women who gave birth at < 34 weeks GA and corticosteroid use

**Countries**	**Hospitals**	**Number of women who ** **gave birth at < 34 weeks**	**Antenatal corticosteroids given to women ** **who gave birth at < 34 weeks**
		**n (%)**	**n (%)**
**Indonesia**	**Overall**	**90 (4)**	**8 (9)**
	Tertiary	69 (7)	6 (9)
	District	21 (2)	2 (10)
**Malaysia**	**Overall**	**51 (2)**	**28 (55)**
	Tertiary 1	32 (3)	19 (59)
	Tertiary 2	19 (2)	9 (47)
**The Philippines**	**Overall**	**48 (2)**	**7 (15)**
	Tertiary 1	15 (1)	0 (0)
	Tertiary 2	33 (3)	7 (21)
**Thailand**	**Overall**	**101 (3)**	**74 (73)**
	Regional	51 (5)	44 (86)
	University	25 (3)	15 (60)
	Provincial	25 (3)	15 (60)

Figures are numbers with percentages rounded to the nearest whole number in brackets.

Demographic information collected about these women included maternal age, any previous birth ≥ 22 weeks, gestational age at time of last corticosteroid injection, if given, whether the pregnancy was singleton or twins, whether they received public or private care, whether they were booked for birth at the hospital and the main reason they were considered at risk of preterm birth (Table 2).

**Table 2:** Characteristics of women receiving/not receiving antenatal corticosteroids with birth 24 to < 34 weeks gestational age

	**Antenatal**	**corticosteroids**			
	**Received**	**Not received**	**Comparative statistic#**	**95% CI**	**p-value**
	**n = 104**	**n = 148**			

Maternal Age* (years)	27.6 (6.8)	28.5 (6.9)	-0.85	-2.59; 0.88	0.34
**Parity **‡					
Previous pregnancies ≥ 22 weeks	47 (45)	86 (58)	0.77	0.60; 0.99	0.04
**Gestational age at last injection**					
< 24 – 27 weeks	20 (19)	-			
28 – 31 weeks	42 (40)	-	N/A	N/A	N/A
32 – < 34 weeks	42 (40)	-			
**Multiple pregnancy**					
Twins	10 (10)	5 (3)	2.84	1.0; 8.1	0.04
**Patient status **‡					
Public	98 (94)	144 (98)	0.96	0.91; 1.01	0.12
**Booked for birth at hospital **‡	81 (79)	136 (92)	0.86	0.77; 0.96	0.01
**Main reasons for risk of preterm birth**					0.01
Preterm labour	42 (40)	36 (24)	-	-	
PPROM	26 (25)	19 (13)	-	-	
Hypertension	18 (17)	18 (12)	-	-	
APH	6 (6)	9 (6)	-	-	
Maternal conditions	4 (4)	6 (4)	-	-	
Multiple pregnancy	2 (2)	2 (1)	-	-	
Fetal concern	1 (1)	8 (5)	-	-	
Other/not recorded	5 (5)	50 (34)	-	-	

Figures are numbers with percentages rounded to the nearest whole number in brackets and with relative risk and 95% confidence intervals as the comparative statistic^#^, or *Mean and standard deviation with mean difference between groups and 95% confidence intervals as the comparative statistic^#^. ‡Outcomes not available for a few women

Health outcomes for all infants exposed to antenatal corticosteroids as well as those who were not were collected including gestational age at birth, birth weight, stillbirth, death prior to discharge, live born babies discharged alive, presence of respiratory distress syndrome, severity of any lung disease, use of oxygen therapy, use and duration of mechanical ventilation (defined as intermittent positive pressure ventilation and/or continuous airways pressure), Apgar scores and length of hospital stay (Table 3).

**Table 3:** Health outcomes for infants exposed to antenatal corticosteroids versus those not exposed

	**Antenatal**	**corticosteroids**			
	**Exposed**	**Not exposed**	**Comparative statistic#**	**95% CI**	**p-value**

***All babies***	***n = 114***	***n = 152***			
Gestational age at birth*‡ (weeks.days)	30.4 (2.6)	29.9 (2.7)	0.47	-0.19; 1.13	0.16
Birth weight*‡ (kg)	1.62 (0.65)	1.48 (0.63)	0.14	-0.02; 0.29	0.08
**Stillbirth**	2 (2)	42 (28)	0.06	0.02; 0.25	0.01
**All deaths (stillbirths/deaths prior to discharge)**	18 (16)	49 (32)	0.49	0.30; 0.79	0.01
**Mode of birth**					0.01
normal vaginal	59 (52)	99 (65)	1	--	--
vaginal breech	4 (4)	19 (13)	0.39	0.14; 1.11	0.06
operative vaginal birth	2 (2)	2 (1)	1.65	0.24; 11.45	0.63
caesarean section	49 (43)	32 (21)	1.85	1.28: 2.67	0.01
***Live born babies***	***n = 112***	***n = 111***			
**Live born babies discharged alive**	96 (86)	103 (93)	0.92	0.84; 1.0	0.05
**Respiratory distress syndrome **‡‡	59 (60)	54 (57)	1.06	0.83; 1.34	0.64
**Severity of lung disease**§					0.01
none	22 (30)	4 (11)	1	--	--
mild	7 (9)	15 (42)	--	--	--
moderate	27 (37)	6 (17)	--	--	--
severe	18 (24)	11 (30)	--	--	
**Use of oxygen therapy¶**	50 (50)	55 (57)	0.86	0.66; 1.12	0.27
**Use of mechanical ventilation¶**	53 (54)	25 (39)	1.38	0.97; 1.98	0.06
**Apgar Score < 7 at 1 min **†	42 (38)	57 (52)	0.73	0.54; 0.98	0.04
**Apgar Score < 7 at 5 min **†	15 (14)	34 (31)	0.44	0.25; 0.75	0.01

Figures are numbers with percentages rounded to the nearest whole number in brackets and with relative risk and 95% confidence intervals as the comparative statistic^#^, or *mean and standard deviation with mean difference between groups and 95% confidence intervals as the comparative statistic^#^. ^§^Severity of lung disease is defined as none (no oxygen required), mild (Oxygen < 40% FiO_2_), moderate (FiO_2 _40–79%), severe (FiO_2 _80% or more). ‡Values are based on 114 in the Exposed Group and 151 in the Not Exposed Group. ‡‡ Values are based on 98 in the Exposed Group and 95 in the Not Exposed Group. ¶Values are based on 101 in the Exposed group and 96 in the Not Exposed Group. ¶P Values are based on 98 in the Exposed Group and 64 in the Not Exposed Group. † Values are based on 111 in the Exposed Group and 110 in the Not Exposed Group.

Maternal health outcomes of all women were collected including use of induction of labour, mode of birth, primary or major postpartum haemorrhage and postpartum pyrexia (Table 4).

**Table 4:** Maternal outcomes for women receiving/not receiving antenatal corticosteroids with birth 24 to < 34 weeks gestational age

	**Antenatal**	**corticosteroids**			
	**Received**	**Not received**	**Relative Risk**	**95% CI**	**p-value**
	**n = 104**	**n = 148**			
**Induction of labour **‡	3 (3)	11 (23)	0.14	0.04; 0.47	0.01
**Primary postpartum haemorrhage (≥ 500 ml) **‡	27 (26)	22 (15)	1.68	1.02; 2.79	0.04
**Major haemorrhage (≥ 1000 ml) **‡	8 (8)	7 (5)	1.57	0.58; 4.19	0.42
**Postpartum pyrexia ≥ 38°C **‡	9 (9)	2 (1)	6.53	1.44; 29.5	0.01

Figures are numbers with percentages rounded to the nearest whole number in brackets and with relative risk and 95% confidence intervals as the comparative statistic. ‡Outcomes not available for a few women.

### Data Analysis

Descriptive statistics assessed the use of antenatal corticosteroids across countries and between hospitals within countries. Difference of means with 95% confidence interval (CI) was used to compare factors measured in continuous data, such as maternal age and birth weight, between the two groups. When the data were skewed, difference of medians with 95% CI was used. Independent t-test or Mann-Whitney U test, where appropriate, was used for significant comparison of the two groups and results were shown as p-values. Relative risk (95% CI) and Chi-square tests were used for comparing the factors measured in categorical data, such as parity and multiple pregnancy between the two groups. The analysis was done using STATA software version 8.0 [10].

### Ethics

The SEA-ORCHID project was approved by the local ethics committees of each hospital and by the ethics committee of the University of Sydney, the administering institution in Australia.

## Results

### Corticosteroid administration in South East Asian countries (Table 1)

Antenatal corticosteroids were not frequently administered to women who gave birth at less than 34 weeks gestation at any of the hospitals audited across the four South East Asian countries. Of the 290 women who gave birth at less than 34 weeks gestation presenting to the audited hospitals, 117 (40%) received antenatal corticosteroids. Thai and Malaysian hospitals showed the highest usage of antenatal corticosteroids for women who gave birth before 34 weeks gestation (73% and 55% respectively). Less than 10% of women who gave birth preterm at Indonesian hospitals were reported to be given antenatal corticosteroids. Hospitals in The Philippines recorded a low rate of antenatal corticosteroid use with one hospital not administering corticosteroids to any women who gave birth preterm.

Dexamethasone was the only antenatal corticosteroid used across the nine hospitals. Repeat courses of antenatal corticosteroids were given to 6% of women.

### Maternal demographics (Table 2)

Overall, women who gave birth at less than 34 weeks gestational age who received antenatal corticosteroids were similar in age to women who did not receive antenatal corticosteroids (mean maternal age 27.6 years (Standard Deviation (SD) 6.8) and 28.5 years (SD 6.9) respectively). Nearly all women in this audit attended a public hospital throughout their pregnancy. Compared with women who did not receive antenatal corticosteroids, women who did were less likely to have had a previous pregnancy ≥ 22 weeks (RR 0.77, [95%CI 0.60, 0.99], p = 0.04), almost three times as likely to have a multiple pregnancy (RR 2.84, [95%CI 1.0, 8.1], p = 0.04) and less likely to be booked for birth at the hospital (RR 0.86, [95%CI 0.77, 0.96], p = 0.01). For women receiving antenatal corticosteroids, most had their last injection after 28 weeks gestation. Similar numbers of women received their last injection between 28 to 31 weeks and between 32 to < 34 weeks gestation (40% and 40%).

There were significant differences in the distributions of the main reason women were considered at risk of preterm birth between the mothers who were administered corticosteroids and those who did not receive them (p = 0.01) (Table 2). More women who received antenatal corticosteroids were in preterm labour, or had preterm prelabour rupture of the membranes or hypertension.

Although health outcomes for the mother and their infants are compared below by whether exposed or not to antenatal corticosteroids, it needs to be remembered these are not randomised groups. Groups may well differ by other factors influencing health outcomes besides the exposure or not to corticosteroids.

### Infant health outcomes (Table 3)

Infants exposed to antenatal corticosteroids were born at a similar gestational age and had similar birth weights to infants who were not exposed. The majority of infants in both groups were born between 28 to < 34 weeks (82% and 80% respectively). Infants exposed to antenatal corticosteroids were less likely to be stillborn (RR 0.06, [95%CI 0.02, 0.25], p = 0.01) and were less likely to die prior to hospital discharge (stillbirth and death prior to discharge combined, RR 0.49, [95%CI 0.30, 0.79], p = 0.01) compared with infants who were not exposed. Live born infants who were not exposed were more likely to be discharged alive than infants who were exposed to antenatal corticosteroids (RR 0.92, [95%CI 0.84, 1.0], p = 0.05].

The distribution of severity of any lung disease was significantly different in infants exposed to antenatal corticosteroids with fewer infants having respiratory problems compared with infants who were not exposed (p = 0.01). Low Apgar scores (< 7) at 1 minute and 5 minutes were less likely for infants exposed to antenatal corticosteroids compared with infants who were not exposed (RR 0.73, [95%CI 0.54, 0.98], p = 0.04 and RR 0.44, [95%CI 0.25, 0.75], p = 0.01 respectively).

The risk of respiratory distress syndrome, use of mechanical ventilation and use of oxygen therapy were not statistically different between the two groups of infants.

### Maternal health outcomes (Table 4)

Significantly fewer women who received antenatal corticosteroids were induced (RR 0.14, [95%CI 0.04, 0.47], p = 0.01) however they were almost twice as likely to have a caesarean section (RR 1.85, [95%CI 1.28, 2.67], p = 0.01) compared with women not who did not receive antenatal corticosteroids. A primary postpartum haemorrhage (≥ 500 ml) was more likely for women who received antenatal corticosteroids (RR 1.68, [95%CI 1.02, 2.79], p = 0.04) and they were over six times more likely to develop a postpartum pyrexia (≥ 38°C) compared with women who did not receive them (RR 6.53, [95%CI 1.44, 29.5], p = 0.01).

## Discussion

An audit by the SEA-ORCHID group across four South East Asian countries found the use of antenatal corticosteroids to be one of the least performed beneficial interventions in the prenatal period at collaborating hospitals within those countries [9]. We therefore conducted this in depth review of antenatal corticosteroid use prior to preterm birth in women presenting to the nine hospitals in four South East Asian countries to compare sociodemographic factors, obstetric history and birth and infant outcomes between women exposed and non-exposed.

This review reported varying uptake of these guidelines into practice both between the nine hospitals in the four South East Asian countries that were audited and between the countries themselves. Within the countries, there was no obvious relationship between the type of hospital and the implementation of the use of corticosteroids, as use varied between hospitals within some countries and was similar between hospitals in other countries. These findings highlight the need to examine the barriers and enablers to adoption of the use of antenatal corticosteroids among maternal and infant health practitioners at the individual hospitals. Furthermore, it illustrates potential benefit in each hospital developing its own locally relevant clinical practice guideline for antenatal corticosteroid administration so as to encourage standardised best practice.

The results of our review show that women from the nine collaborating hospitals who received antenatal corticosteroids were more likely to be experiencing their first pregnancy and have a multiple pregnancy but were less likely to be booked at the hospital for birth. There remains doubt about the use of antenatal corticosteroids in multiple pregnancies. One study has reported similar risks of death or major morbidity between singleton and multiple infants [11] while other studies suggest that antenatal corticosteroid therapy does not significantly reduce the incidence of respiratory distress syndrome in multiple gestations [12-15]. Further analysis of health outcomes of infants from multiple pregnancies need to be analysed to provide evidence either for or against this practice [4].

Our review showed that in the nine hospitals in the four South East Asian countries audited, over 80% of corticosteroids administered were given when women were over 28 weeks gestation. Gestational age at which antenatal corticosteroids should be given has long been a topic of debate. An earlier systematic review showed limited evidence of benefit if given at less than 28 weeks [16] although the updated systematic review shows respiratory distress syndrome is reduced when corticosteroids are given in multiple doses with the first injection between 26 to 29.9 weeks [4].

The type of antenatal corticosteroid used between countries and between hospitals within countries did not differ, with dexamethasone the only corticosteroid used. It is recognised that the frequency of respiratory distress syndrome is decreased by both betamethasone and dexamethasone [4], although there is still uncertainty concerning which corticosteroid provides better health outcomes [17].

The reason as to only dexamethasone being used in the South East Asian countries audited is most likely due to financial reasons, given that dexamethasone is the less expensive of the two corticosteroids. As a result of this, dexamethasone is often simply the only drug available in these developing countries.

Limited use of repeat steroids was reported for all four South East Asian countries. Evidence for the use of a single course of antenatal corticosteroids for women at risk of preterm birth has been acknowledged and accepted and it has become recommended standard care [7,18,19]. At the time of this audit, evidence regarding the effects of repeat courses of antenatal corticosteroids was still unclear [20].

The published systematic reviews on antenatal corticosteroids papers [4] report positive health outcomes for mothers and infants. The few negative health outcomes seen in our audit are likely to reflect selection bias due to the use of selective, non randomised groups.

Based on abundant evidence suggesting that antenatal corticosteroids positively influence infant survival for preterm birth, and the audit result of varying uptake of this intervention in the participating hospitals, it is obvious that barriers to uptake of practice, in particular access to evidence-based practice guidelines and recommendations need to be examined at the individual institutions. As part of the SEA-ORCHID project we plan to conduct a survey of evidence-based practice knowledge and clinical change among maternal and infant health practitioners in South-East Asia to explore this issue.

A similar intervention undertaken in Thailand increased the rate of use of antenatal corticosteroids for women who gave birth preterm (< 34 weeks gestation) from a baseline rate of 41% to 73% after evidence became available and was disseminated [21]. Prior to this intervention an increase from 33% to 58% for the rate of antenatal corticosteroid administration in the US was achieved after distribution of information. A further increase to 68% was achieved after further knowledge of evidence distribution [22].

## Conclusion

This audit across four South East Asian countries indicates that rates of use of antenatal corticosteroids are varied in the hospitals participating in the SEA-ORCHID project. Antenatal corticosteroids given to women prior to preterm birth had significant health benefits for their babies. The findings of this review highlight an important gap in best practice recommendations that could be improved with relatively low cost and with great benefit to neonatal outcomes in resource poor countries. Barriers to uptake and implementation of evidence-based practice guidelines need to be evaluated and addressed and form part of the ongoing SEA-ORCHID project [8].

## Competing interests

The authors declare that they have no competing interests.

## Authors' contributions

PL, ML, SM and CC contributed to the design of the study. PP cleaned and analysed the data and all authors contributed to the interpretation of data. ME prepared the first draft of the paper. All authors commented on each draft of the paper.

## Pre-publication history

The pre-publication history for this paper can be accessed here:


